# Insights into the tissue repair features of MAIT cells

**DOI:** 10.3389/fimmu.2024.1432651

**Published:** 2024-07-16

**Authors:** Mengge Gao, Xiaosu Zhao

**Affiliations:** ^1^ Peking University People's Hospital, Peking University Institute of Hematology, National Clinical Research Center for Hematologic Disease, Beijing Key Laboratory of Hematopoietic Stem Cell Transplantation, Beijing, China; ^2^ Research Unit of Key Technique for Diagnosis and Treatments of Hematologic Malignancies, Chinese Academy of Medical Sciences, Beijing, China; ^3^ Collaborative Innovation Center of Hematology, Peking University, Beijing, China

**Keywords:** mucosal-associated invariant T cell, tissue repair, chemotaxis, tissue phenotype, immunoregulatory effects

## Abstract

Mucosa-associated invariant T (MAIT) cells are a subset of innate-like non-conventional T cells characterized by multifunctionality. In addition to their well-recognized antimicrobial activity, increasing attention is being drawn towards their roles in tissue homeostasis and repair. However, the precise mechanisms underlying these functions remain incompletely understood and are still subject to ongoing exploration. Currently, it appears that the tissue localization of MAIT cells and the nature of the diseases or stimuli, whether acute or chronic, may induce a dynamic interplay between their pro-inflammatory and anti-inflammatory, or pathogenic and reparative functions. Therefore, elucidating the conditions and mechanisms of MAIT cells’ reparative functions is crucial for fully maximizing their protective effects and advancing future MAIT-related therapies. In this review, we will comprehensively discuss the establishment and potential mechanisms of their tissue repair functions as well as the translational application prospects and current challenges in this field.

## 1 Introduction

Over the past three decades, innate-like lymphocytes such as mucosa-associated invariant T (MAIT) cells, NKT cells, and γδ T cells have emerged as a unique class of immune cells that bridge innate and adaptive immunity. These cells acquire their effector functions during development and stably reside in peripheral tissues, ready to respond immediately upon recognizing primary antigens (1–5). MAIT cells (CD3+CD161hiVα7.2+) express a conserved TCRα chain Vα7.2-Jα33 (corresponding to mouse Vα19–Jα33) (4–6), primarily recognizing monomorphic MHC-1-related molecule (MR1). This recognition allows them to respond to small molecule antigens such as 5-(2-oxopropylideneamino)-6-D-ribitylaminouracil (5-OP-RU) and 5-(2-oxoethylideneamino)-6-D-ribitylaminouracil (5-OE-RU), derived from the riboflavin metabolic pathway produced by the microbiota (7, 8). Consequently, MAIT cells are considered particularly dependent on the microbiota. In germ-free (GF) mice, the frequency of MAIT cells is significantly lower compared to those raised under specific-pathogen-free (SPF) conditions (2, 9, 10).

In recent years, transcriptomic technologies have greatly advanced the exploration of MAIT cell heterogeneity across various contexts. Beyond their well-documented anti-infective activities against bacteria and viruses (11–17), recent studies have highlighted the crucial role of MAIT cells in tissue homeostasis and repair (2, 18–22). Current research indicates that MAIT cells contribute to tissue repair primarily through homing and chemotactic migration. These cells interact with mucosal microbiota or other immune cells, leading to the inhibition of inflammatory responses or the secretion of repair mediators. This process maintains mucosal barrier function and facilitates tissue repair. The following discussion provides a detailed examination of the significant properties of MAIT cells in tissue repair and their potential future applications.

## 2 Tissue phenotype and targeted chemotaxis

MAIT cells are enriched in human intestines (10%) (2, 23–27), lungs (3%) (28), liver (10%-40%) (29), skin (0.5%-2%) (8) (Above frequency as % of total T cells or αβT cells.), as well as urogenital tract (30–32). They constitute a crucial subset of immune cells within mucosal tissues. These tissues are also colonized by diverse microbial communities, which play a significant role in the development, differentiation, and activation of MAIT cells (2, 15–18, 33). MAIT cells are classified as MAIT1 and MAIT17 based on the expression of transcription factors T-bet and RORγt, respectively. However, in humans, MAIT cells exhibit a mixed gene expression pattern without distinct functional subsets (34, 35). In naive SPF mice, tissue-resident MAIT cells are predominantly RORγt+T-bet− (MAIT17), producing IL-17A, while a minor population of circulating MAIT cells is T-bet+RORγt− (MAIT1), producing IFN-γ. Following intranasal infection with pathogens such as Salmonella or Legionella, an increase in RORγt+T-bet+ lung MAIT cells is observed. This suggests that the diverse stimuli received by human MAIT cells may explain some interspecies differences (36, 37). Additionally, in the female reproductive tract and oral mucosa, barrier MAIT cells predominantly exhibit a mature CD4−CD8− MAIT17 phenotype with higher RORγt and lower T-bet expression (23, 38, 39). The transcriptional profile of mouse tissues reveals specific differences with lung tissue tending towards MAIT17 and liver tissue towards MAIT1 (34), likely due to microbial exposure or other environmental factors.

The enrichment of MAIT cells within tissues is primarily due to their high surface expression of various chemokine receptors and tissue residency markers, such as CD69 and CD103 (23, 24, 29, 40). The tissue homing process is driven by the master transcription factor promyelocytic leukemia zinc finger protein (PLZF), which decreases the expression of Klf2 and its target CD62L (7, 33, 40–45). Within the thymus, MAIT cells acquire distinctive tissue residency traits (33, 41–44) and exhibit high levels of CCR2, CCR8, and CXCR6 expression. Upon exiting the thymus, these cells are directed towards different non-lymphoid tissues, with tropism potentially varying among cell subsets (29, 33–35, 46). MAIT1 cells are primarily localized in the spleen, lymph nodes, and liver, whereas MAIT17 cells are enriched in barrier tissues such as the lungs, skin, and intestines (1, 33–35, 41–46), possibly driven by differentiation programs involving the expression of T-bet and RORγt and surface chemokine receptor profiles.

Single-cell sequencing data reveal that circulating MAIT cells express various chemokine receptor receptors, including CCR2 (associated with inflammation tissue infiltration), CCR5, CCR8 (potentially targeting skin and lung tissues), CXCR3 (homing to inflamed tissues), CCR6 (tropism towards skin, gut, and brain), and CXCR6 (tropism towards gut-liver interfaces) (23, 28, 29, 33, 47). CXCR3 is predominantly expressed in the MAIT1 subset, facilitating their migration towards sites of infection or inflammation (35, 45, 46, 48), while MAIT17 cells exhibit high expression of CCR6 and CXCR6 (35, 45, 46, 49), associated with mucosal migration to organs such as the liver (29, 50–52), intestines (53), and lungs (28). This diversity leads to heterogeneity in MAIT cell phenotypes and subsets between blood and tissues, as well as within tissues themselves. However, it is currently unclear whether tissue-resident mucosal MAIT cells can exit mucosal tissues and recirculate.

**Figure 1:**
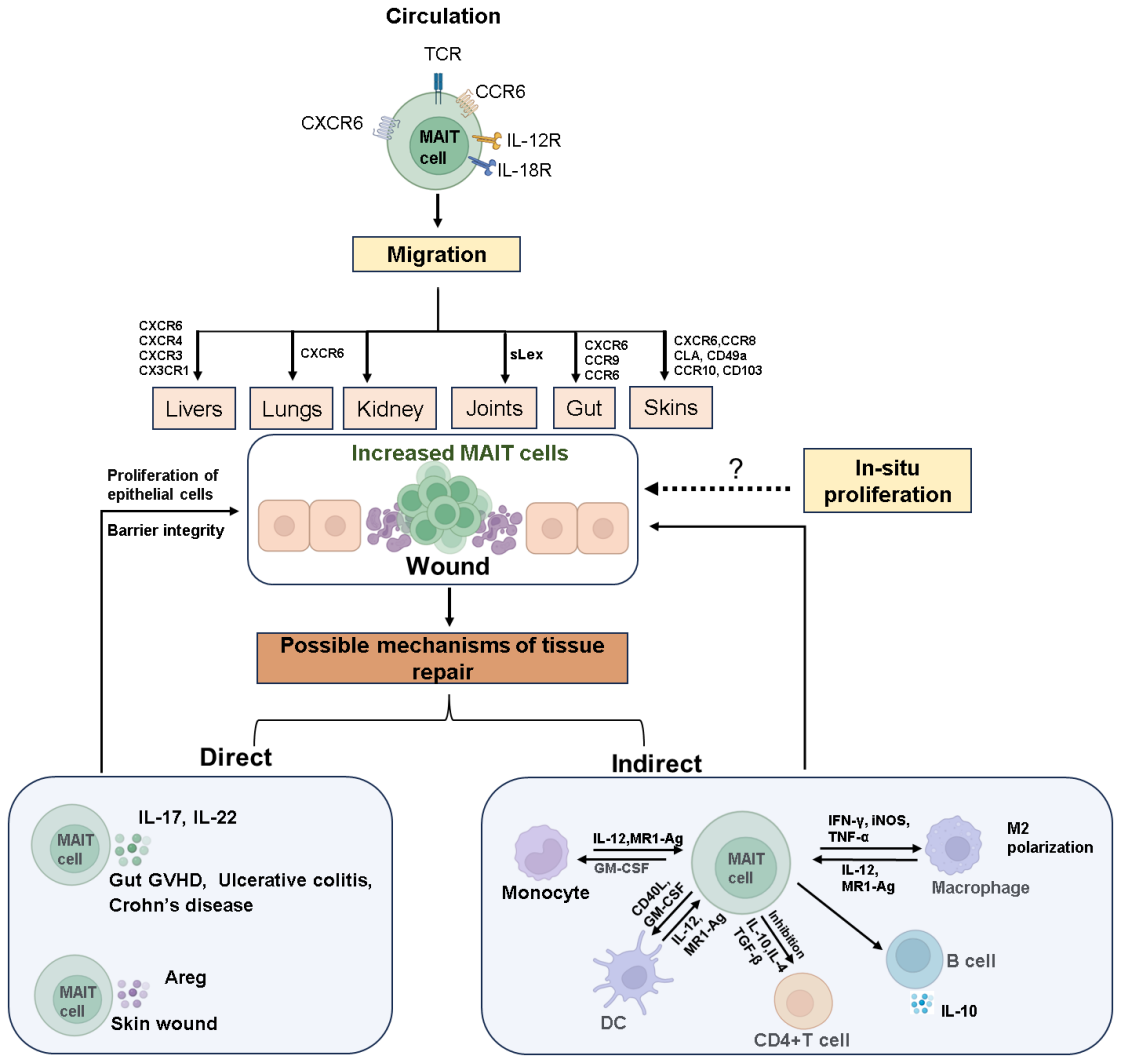
The mechanisms of MAIT cell accumulation at wound sites and their role in tissue repair. Circulating MAIT cells express various cytokine and chemokine receptors, enabling them to target and migrate to different tissues. Upon tissue damage, MAIT cells accumulate at the site of wound through chemotaxis or *in-situ* proliferation. The invasion of mucosal microbes or inflammatory stimuli rapidly activates MAIT cells, prompting them to secrete tissue repair factors such as IL-17, IL-22, and Areg. These cells also interact with other immune cells to facilitate the repair of damaged tissue. The dashed arrows in the Figure indicate that these processes require further experimental evidence for support. Created with Biorender.com.

Under pathological conditions such as mucosal inflammation or tissue injury, MAIT cell numbers at the site of pathology rapidly increase through both *in-situ* proliferation and recruitment from circulation (**Figure 1**). For instance, in pulmonary infection with *Francisella tularensis live vaccine strain (LVS)*, CXCR6 facilitates long-term retention of MAIT cells in affected tissues, where they proliferate locally rather than being recruited from secondary lymphoid tissues (28). Conversely, studies in mice have shown that increased MAIT cells at the skin wound recruit from the circulation system in a CXCR6-CXCL16-dependent manner and initiate wound repair functions (54). Our studies using NOD-SCID-IL-2Rg−/−(NSG) mouse transplant models simulated the dynamic distribution of MAIT cells and indicated that circulating MAIT cells may recruit to intestinal tissues via CXCR6 (55).

MAIT cell migration and chemotaxis are prominent in clinical diseases. In immune-mediated diseases like primary Sjögren’s syndrome (pSS) (56), primary biliary cholangitis (PBC) (57), inflammatory bowel disease (IBD) (58–61), rheumatoid arthritis (RA) (62), and type 1 diabetes (T1D) (63–65), the chemokine and cytokine milieu in inflamed tissues drives MAIT cell via various chemokine receptors such as CCR9, CXCR5, CXCR4, CXCR3, CCR6, and CCR10 (**Figure 1**). For instance, in PBC, MAIT cells accumulate in the liver through CXCL12-CXCR4 chemotaxis (57), while in IBD, chemokines like CCL20, CXCL10, CXCL16, and CCL25 might guide MAIT cells to the inflamed gut (58). In newly diagnosed T1D children, the frequency of blood MAIT cells positively correlates with CCR6 expression, suggesting a role for CCR6 in their migration to inflamed tissues. CCR6-expressing cells recognize CCL20 and β-defensins, which are elevated in the pancreas and intestines of diabetic patients and mouse models (66, 67). Other homing receptors like CCR10, CD49a, CD103, and skin lymphocyte-associated antigen may direct MAIT cells to dermatitis herpetiform lesions (24, 38, 68, 69). Additionally, in crescentic glomerulonephritis (cGN), MAIT17 cells interact with pro-inflammatory myeloid cells in the kidney via the CXCR6–CXCL16 axis, suppressing their tissue-destructive capabilities (70).

In some diseases like systemic lupus erythematosus (SLE), Type 2 diabetes, and obesity, MAIT cells exhibit an activated and exhausted state (71–73). The decreased frequency of blood MAIT cells may reflect their migration to inflamed tissues or exhaustion upon activation. As inflammation progresses to chronic stages (74), other unidentified signals might sustain the accumulation and survival of MAIT cells in specific inflamed tissues. Although specific subsets of MAIT cells that preferentially traffic to different tissues have not been fully elucidated, their abundant expression of chemokine receptors and migratory capacity are crucial for their roles in tissue homeostasis and repair.

## 3 Maintenance and triggering of tissue-protective functions

Similar to iNKT cells, MAIT cells can be activated via their TCR, recognizing microbial riboflavin metabolism intermediates like 5-OP-RU bound to MR1, or independently by pro-inflammatory cytokines (8, 18, 19, 75–77). These activation mechanisms differ different kinetics: TCR-mediated activation leads to rapid production of a broad array of pro-inflammatory cytokines and chemokines, including IL-1A, IL-1B, IL-2, IL-22, GM-CSF, CCL3, CCL4, and CCL20, with rapid IFN-γ release within 6 hours. In contrast, MAIT cells activation by IL-12 and IL-18 primarily induces IFN-γ production at 20-24 hours (18, 19, 75, 76).

Given their strategic location in mucosal tissues and activation by microbial metabolites, MAIT cells play crucial roles in maintaining tissue homeostasis. Previous studies indicate the response of unconventional H2-M3-restricted Tc17 cells to commensal S epidermidis, suggesting a role in regulating tissue homeostasis similar to other innate-like T cells involved in barrier surface homeostasis (36, 78, 79). Supported by a diverse gut microbiome, MAIT cells, along with Vδ2 unconventional T cells, are supported by a diverse gut microbiome and associated with favorable prognosis in patients post-allogeneic hematopoietic cell transplantation (HCT) (79). In gut graft-versus-host disease (GVHD) mouse models, Mr1-deficient mice lacking MAIT cells exhibit reduced gut microbial diversity, akin to IL-17A-deficient animals (22), highlighting the protective role of MAIT cells in intestinal inflammation. Recent findings by El Morr et al. demonstrated that during intestinal inflammation, MAIT cells detect microbiota-derived metabolites and promote tissue repair. Under normal conditions, these metabolites from aerotolerant bacteria in the colonic mucosa activate MAIT cells. During inflammation, increased production of these ligands crosses the intestinal barrier, activating MAIT cells to express repair genes and produce barrier-enhancing mediators, facilitating colitis resolution (80). Understanding these interactions between MAIT cells and the microbiome is crucial for elucidating their role in maintaining tissue homeostasis.

Transcriptional analyses in both mice and humans reveal a strong enrichment of tissue repair signatures in MAIT cells, particularly the MAIT17 subset [18,19,43,80]. In mucosal or wound tissues, the predominant phenotype of MAIT cells is MAIT17 (8), attributed to: ① Under steady-state conditions, the microbial environment of mucosal barriers promotes the differentiation of MAIT17 and the maintenance of its repair functional programs; ② Under stress, recruitment of MAIT17 cells from circulation to wounds becomes predominant. The functional effects of these transcriptional programs have been demonstrated both *in vivo* and *in vitro*. Using an *in vitro* wound healing assay with the Caco2 intestinal epithelial cell line, it was found that MAIT cells trigger inducible tissue repair programs in an MR1-dependent manner and accelerate wound closure in this system (19). This was demonstrated in mouse models, where Mr1-/- NOD mice exhibited increased intestinal permeability compared to Mr1+/+ NOD mice, indicating a protective role of MR1-mediated MAIT cells in maintaining intestinal homeostasis (60). Similarly, *in vitro* human cholangiocyte cell line H69 wound healing assay, MAIT cells showed wound healing characteristics dependent on MR1-antigen-TCR interaction conduction (81). Direct application of 5-OP-RU on injured skin was also sufficient to expand MAIT cells and accelerate tissue repair in mice (10). However, in a recent human-like mouse model of full-thickness skin excision, MAIT cells in the skin express tissue repair programs in a steady-state, but the recruitment and tissue repair functions of MAIT cells do not depend on MR1-mediated antigen presentation (54). The potential reasons for this contradictory result include, firstly, the need for additional signals to initiate and amplify tissue repair programs, apart from solely relying on MR1 molecules (18, 19, 82–86). Secondly, pro-inflammatory cytokines, along with TCR signaling, trigger robust and sustained effector functions of MAIT cells, which are not only reflected in the upregulation of repair factors but also in the release of various cytokines such as IFN-γ and TNF-α (18, 19, 82–86), affecting the inflammation response and immune cell activity, thereby helping to regulate the level of inflammation during tissue injury and repair processes. Lastly, classical TCR signaling through CD3/CD28 stimulation also increases the expression of mucosal protective factors such as IL-17A (84), although possibly to a lesser extent than activation via the MR1 pathway.

In summary, tissue repair is a complex process regulated by various factors. When MAIT cells are stimulated through TCR without pro-inflammatory cytokines, they secrete epithelial repair factors, contributing to the maintenance of homeostatic barriers. However, in the presence of cytokines accompanying wounds or inflammation, MAIT cells initiate additional anti-infective responses, aiding in emergency barrier repair and bolstering host defense mechanisms (19). This dual role underscores the versatility and adaptability of MAIT cells in maintaining tissue integrity and responding to different challenges.

## 4 Immunoregulatory effects

Upon activation at mucosal or pathological sites, MAIT cells exert their effects through two pathways (**Figure 1**): direct action by secreting tissue repair factors to promote wound healing and indirect action by influencing other immune cell subsets through immunoregulation.

Transcriptomic studies of MAIT cells from human and mice have revealed shared expression of genes involved in tissue protection and repair. These genes include immune genes (TNF, PTGES2, TGFB1, CCL3, HMGB1), proteases (Furin, MMP25), growth factors (GM-CSF, M-CSF, PDGFB, LIF), and angiogenic genes (HIF1A, VEGFB) (10, 18–20). In the latest study by Sayaf. K et al., the MAIT cell stimulation leads to the production of growth factors, potentially mediating their critical role in tissue repair and regeneration following injury through the VEGF-VEGFR2 signaling pathway (87).

The roles of IL-17 (22, 88, 89) and amphiregulin (Areg) (54, 90) in tissue repair have been well studied. Areg is an epidermal growth factor-like molecule, mediates keratinocyte proliferation (90). During the skin injury repair, MAIT cells induce wound healing by secreting Areg (54). MAIT cells also play a crucial role in maintaining intestinal mucosal integrity by producing IL-17 and IL-22 (21, 22). In Type 1 Diabetes (T1D), a reduction in IL-17 and IL-22 production by MAIT cells leads to compromised mucosal barrier integrity and increased intestinal permeability (23, 63–65, 89, 91–93). This can trigger local intestinal inflammation and facilitate the translocation of bacterial compounds to the liver or pancreatic lymph nodes, exacerbating autoimmunity and disease progression (63, 64). In a mouse transplant model, residual intestinal MAIT cells from recipient mice secrete IL-17 to maintain intestinal integrity and suppress gut GVHD (22, 94). IL-22 promotes the survival and proliferation of epithelial cells, while IL-17 regulates tight junction proteins to prevent excessive barrier permeability during epithelial injury (89, 95, 96). Additionally, MAIT cell-deficient mice have been shown to develop intestinal leakage, though underlying mechanisms require further investigation (63).

MAIT cells also maintain tissue homeostasis at the meningeal barrier by expressing antioxidant molecules such as Selenop and Fth1, which enhance the expression of cell adhesion molecules like E-cadherin and Claudin11, contributing to meningeal barrier integrity (97). These findings highlight the crucial protective role of tissue repair factors secreted by MAIT cells in mucosal homeostasis and repair.

In addition to direct tissue repair, MAIT cells exert immunomodulatory effects indirectly. They trigger dendritic cells (DCs) maturation in a CD40L and GM-CSF-dependent manner, inducing anti-inflammatory macrophage polarization and promoting B cell differentiation and antibody production (62, 98–100). In experimental autoimmune encephalomyelitis (EAE), MAIT cells inhibit disease development by modulating pro-inflammatory molecules and promoting IL-10 production by B cells, reducing disease severity (6). Activated MAIT cells can induce differentiation of monocytes/macrophages into an M2 phenotype *in vitro* (50) and promote long-term survival of neutrophils and their differentiation into APC-like neutrophils via TNF, IFN-γ, and GM-CSF mediation (101–103). By modulating neutrophils and DCs, MAIT cells increase the number of effector and memory conventional CD4+ and CD8+ T cells at infection sites and new arrivals (62, 101). They may also recruit other tissue repair immune cells such as macrophages by producing chemotactic factors like CCL3 (19, 104, 105). Additionally, MAIT cells can inhibit CD4+ T cell proliferation *in vitro* (88–90). In an allogeneic reaction *in vitro* model, MAIT cells effectively control or delay the occurrence of GVHD through immunosuppressive effects (106–108), correlating with reduced infiltrating human T cell numbers, proliferation, and effector function in diseased mouse tissues, along with reduced circulating levels of IFN-γ and TNF-α and increased levels of IL-10 (108). In summary, MAIT cells can engage in repair or antimicrobial responses within mucosal tissues through cross-talk with other immune cells. However, detailed studies using mucosal tissue samples, such as those from the gastrointestinal tract, are necessary to better understand this phenomenon.

Recent evidence shows that MAIT cells play a significant role in the immune response to SARS-CoV-2. In COVID-19 patients, circulating MAIT cells significantly decrease and become enriched in the airways (109–114). The remaining blood MAIT cells are activated, with increased CD69, CD38, HLA-DR, CD56, and granzyme B expression, along with decreased CXCR3 expression. Airway MAIT produce IL-17A and TNF, associated with chemokines like CXCL10 and CX3CL1 (109, 112). Regarding clinical outcomes, current research shows inconsistent results. Parrot T et al. found that high CD69 and low CXCR3 expression on MAIT cells are associated with mortality (112). In contrast, research by Jouan Y et al. indicated that CD69 expression on MAIT and iNKT cells at admission is associated with improved oxygenation on day 7 and increased discharge from intensive care by day 15 (113), suggesting a beneficial role for MAIT cells in COVID-19. The data suggest MAIT cell dual role in contributing to inflammation and aiding in disease resolution. Additionally, during SARS-CoV-2 infection, SARS-CoV-2-infected macrophages can activate MAIT cell through MR1-dependent degranulation or the cytokine IL-18 (114). In severe COVID-19 patients, IL-10 suppresses monocyte HLA-DR expression, leading to MAIT cell dysfunction (115). Overall, the balance between protective and pro-inflammatory roles of MAIT cells, and their potential tissue repair function, remains unclear, but their distinct changes during SARS-CoV-2 infection underscore their significant role.

In summary, in chronic pathological conditions like viral infections and autoimmune, inflammatory, and metabolic diseases, MAIT cells can exert pathogenic effects through sustained inflammation and cytotoxicity. The balance between their pathogenic and protective roles may depend on factors such as activation status, tissue localization, cytokine profile, and disease chronicity (24). In conditions of chronic inflammation, tissue-resident MAIT cells may lose their homeostatic function, contributing to barrier integrity disruption.

## 5 Future outlook and outstanding issues

MAIT cells’ significant roles in maintaining homeostasis and promoting tissue repair in the intestinal mucosa, meningeal barrier, and skin have garnered increasing attention. Beyond their well-established antimicrobial responses, there is a growing interest in their regulatory role in wound healing. From a translational medicine perspective, efforts are being made to utilize the unique homeostatic functions of MAIT cells to address chronic inflammation and restore tissue integrity. For example, chronic skin wounds such as leg ulcers, sacral pressure sores, or burns could potentially benefit from local reintroduction of symbiotic organisms capable of synthesizing riboflavin or the application of synthetic MAIT cell ligands (98).

Recent research indicates that MAIT cells lack alloreactive potential (116, 117), suggesting potential for developing universal MAIT cell adoptive therapy to overcome HLA disparities. Strict regulation of cell surface MR1 during allo-HSCT can minimize the risk of off-target effects of MAIT cells (75, 118). Additionally, due to their natural tropism for mucosal tissues, MAIT cells can effectively target mucosal tissues such as the gut and skin (118, 119). This highly conservative MR1 regulation makes allogeneic MAIT cell transplantation less likely to induce severe GVHD. Leveraging the effector or tissue repair/regulatory properties of MAIT cells in adoptive immunotherapy offers novel clinical strategies for treating GVHD. MAIT cell transplantation has been preliminarily validated in mouse models and could readily transition to clinical trials. Overall, the tissue repair and regulatory functions of MAIT cells open new avenues for clinical benefits. Furthermore, these characteristics make MAIT cells highly promising candidates for engineered chimeric antigen receptor (CAR)-MAIT therapy.

However, challenges remain, particularly in obtaining human mucosal tissue and developing *ex vivo* research models that accurately simulating *in vivo* disease environments. This limitation hinders comprehensive investigations into the reparative mechanisms of MAIT cells across different diseases or microenvironments. At present, MAIT cells can toggle between pro-inflammatory and anti-inflammatory, or pathogenic and reparative functions, depending on tissue localization and disease types (21, 57, 59, 60). Therefore, several potential obstacles must be addressed before successful clinical translation. Firstly, in-depth exploration of MAIT cell tissue localization and maintenance of repair functions, as well as the conditions or mechanisms triggering them, is crucial for developing targeted therapies for wounds. Secondly, studying the functions and tissue localization of different MAIT cell subtypes is necessary to determine the optimal cell type for transplantation. Finally, a thorough investigation into the antigens or stimuli present in healthy and different diseased tissue microenvironments is essential to understand how these conditions affect MAIT cell functional differentiation, thereby guiding the development of MAIT cell-related therapies conducive to disease recovery in various scenarios. It is noteworthy that understanding the degree of crosstalk between MAIT cells and other immune/non-immune cells and symbiotic microorganisms under normal conditions or when activated in disease microenvironments is also necessary.

To advance the study of MAIT cell reparative functions, it is imperative to develop novel *ex vivo* research models. For instance, fully utilizing current organoid culture techniques may be beneficial. Organoids, self-organizing, miniaturized organs derived from a series of stem cells, replicating key structural and functional features of their *in vivo* counterparts (109, 120–124). Constructing 3D models of human *ex vivo* skin, gastrointestinal tract, and other organoids, and exploring the interaction between co-culture systems and immune cells, can provide deeper insights into the functional aspects of MAIT cells in organoid models under different disease conditions. This includes migration, cytokine production, tissue repair, and antimicrobial activity (109). Additionally, these models can help describe the crosstalk between MAIT cells and resident mucosal immune cells and the consequent impacts on mucosal integrity and immunity. Recently, this technology has been applied to study MAIT cells in acute intestinal inflammation (125). In patient-derived appendiceal organoid (PDAO) models, circulating MAIT cells upregulated chemokine receptors and showed enhanced *E. coli*-pulsed PDAO infiltration in a CCR1-, CCR2-, and CCR4-dependent manner (125). This serves as an excellent preclinical model for investigating the roles of MAIT cells in mucosal organs. It is anticipated that this approach will be applied to other clinical disease models involving MAIT cells in the future, further advancing the clinical translation of MAIT cell research.

## 6 Conclusions

In conclusion, this review outlines the tissue repair effects of MAIT cells and their involvement in various diseases, summarizing current MAIT-related researches. We envisage using organoid models to enhance understanding the interactions between MAIT cells and different disease microenvironments. This approach provides a more robust theoretical basis and preclinical research means for the development of MAIT cell therapies.

## Author contributions

GM: Methodology, Writing – original draft. ZX: Funding acquisition, Methodology, Supervision, Writing – review & editing.
